# Intravenous Immunoglobulin G Suppresses Heat Shock Protein (HSP)-70 Expression and Enhances the Activity of HSP90 and Proteasome Inhibitors

**DOI:** 10.3389/fimmu.2020.01816

**Published:** 2020-08-13

**Authors:** Richard J. Jones, Ram K. Singh, Fazal Shirazi, Jie Wan, Hua Wang, Xiaobin Wang, Min Jin Ha, Muhamed Baljevic, Isere Kuiatse, Richard E. Davis, Robert Z. Orlowski

**Affiliations:** ^1^Department of Lymphoma and Myeloma, The University of Texas MD Anderson Cancer Center, Houston, TX, United States; ^2^The Urology Department, ShengJing Hospital, China Medical University, ShenYang, China; ^3^Department of Biostatistics, The University of Texas MD Anderson Cancer Center, Houston, TX, United States; ^4^Department of Internal Medicine, University of Nebraska Medical Center, Omaha, NE, United States; ^5^Department of Experimental Therapeutics, The University of Texas MD Anderson Cancer Center, Houston, TX, United States

**Keywords:** IVIgG, HSP70, heat shock response, bortezomib, extracellular vesicles

## Abstract

Intravenous immunoglobulin G (IVIgG) is approved for primary immunodeficiency syndromes but may induce anti-cancer effects, and while this has been attributed to its anti-inflammatory properties, IgG against specific tumor targets may play a role. We evaluated IVIgG alone, and with a Heat shock protein (HSP)-90 or proteasome inhibitor, using multiple myeloma and mantle cell lymphoma (MCL) cells *in vitro*, and with the proteasome inhibitor bortezomib *in vivo*. IVIgG inhibited the growth of all cell lines tested, induced G_1_ cell cycle arrest, and suppressed pro-tumor cytokines including Interleukin (IL)-6, IL-8, and IL-10. Genomic and proteomic studies showed that IVIgG reduced tumor cell HSP70-1 levels by suppressing the ability of extracellular HSP70-1 to stimulate endogenous *HSP70-1* promoter activity, and reduced extracellular vesicle uptake. Preparations of IVIgG were found to contain high titers of anti-HSP70-1 IgG, and recombinant HSP70-1 reduced the efficacy of IVIgG to suppress HSP70-1 levels. Combining IVIgG with the HSP90 inhibitor AUY922 produced superior cell growth inhibition and correlated with HSP70-1 suppression. Also, IVIgG with bortezomib or carfilzomib was superior to each single agent, and enhanced bortezomib's activity in bortezomib-resistant myeloma cells. Moreover, IVIgG reduced transfer of extracellular vesicles (EVs) to cells, and blocked transfer of bortezomib resistance through EVs. Finally, IVIgG with bortezomib were superior to the single agents in an *in vivo* myeloma model. These studies support the possibility that anti-HSP70-1 IgG contained in IVIgG can inhibit myeloma and MCL growth by interfering with a novel mechanism involving uptake of exogenous HSP70-1 which then induces its own promoter.

## Introduction

Immunoparesis with suppression of uninvolved immunoglobulins is a well-recognized feature of multiple myeloma (MM) and has been associated with an inferior prognosis. For example, studies have linked immunoparesis with an increased risk of progression to MM from precursor states (1–6). Similarly, several studies suggest immunoparesis in newly diagnosed patients is connected with a worse progression-free and overall survival (7, 8), while immunoglobulin recovery after high-dose therapy confers a good prognosis (9, 10). These relationships may be due to the association of immunoparesis with disease burden and an increased risk of infection (11–13). The latter has prompted consideration of immunoglobulin replacement, and two older randomized studies found this reduced serious infections (14, 15). However, as antibiotic prophylaxis was not used in these trials and myeloma care has changed since then, current consensus guidelines do not routinely recommend prophylactic gamma-globulin (16).

Beyond its use to replace endogenous gamma-globulins in primary immune deficiency patients (17), intravenous gamma-globulin (IVIgG) is used in auto-immune and inflammatory conditions (18). Its immune modulating properties may be due to several mechanisms, including modifying complement activation, suppressing idiotypic antibodies, saturating Fc receptors, and quelling inflammatory mediators (18). At the molecular level, IVIgG may inhibit Nuclear factor kappa-B (NF-κB) activation (19–22), and impact on Mitogen-activated protein kinase (MAPK) pathways (21, 23), Tumor necrosis factor-α (TNF-α) (23), and Toll-like receptor signaling (24–26).

The ability of IVIgG to suppress inflammation suggests it has a role in suppressing malignant cell growth given the relationship between inflammation and cancer. In case reports, patients with Kaposi's sarcoma, thyroid cancer, peripheral nerve sheath tumors, and pancreatic cancer experienced remissions or tumor growth delay after receiving IVIgG (27, 28). Furthermore, a trial in patients with advanced melanoma reported a therapeutic effect of the anti-inflammatory, 2 g/kg IVIgG dose (29). Moreover, some reports suggested IVIgG may enhance endogenous immunoglobulin production (30), and we therefore hypothesized that this could increase proteotoxic stress, a key mechanism of action for proteasome inhibitors in myeloma (31).

Given this rationale, we examined the potential anti-myeloma effect of IVIgG, a polyclonal product pooled from 1,000s to 100,000 blood and plasma donors (32) containing 1,000 of antibodies against an array of targets (33). IVIgG induced an anti-proliferative and pro-apoptotic effect in myeloma and mantle cell lymphoma (MCL) cell lines. This was accompanied by a reduction of the abundance of Heat shock protein (HSP)-70 protein, and *HSP70* promoter activity inhibition. The latter occurred in association with blockade of uptake of extracellular vesicles by the IVIgG preparation, and interruption of an autocatalytic loop whereby extracellular HSP70 stimulated its own intracellular transcription. Finally, IVIgG enhanced the anti-myeloma activity of an HSP90 and a proteasome inhibitor, both of which strongly induce anti-apoptotic HSP70, and an IVIgG/bortezomib combination enhanced *in vivo* anti-myeloma efficacy. These studies shed new light on the mechanisms of action of IVIgG, and on its potential applicability to myeloma patients in particular.

## Materials and Methods

### Reagents

IVIgG and rituximab (RTX) were from The University of Texas MD Anderson Cancer Center Pharmacy (Houston, TX), and IVIgG was dialyzed against phosphate-buffered saline (PBS) to remove L-proline. AUY922, bortezomib (BZB), and carfilzomib (CFZ) were from Selleck Chemicals (Houston, TX), recombinant human HSP70-1 protein was from StressMarq Biosciences (British Columbia, Canada), and bovine serum albumin (BSA) was from Sigma-Aldrich (St. Louis, MO).

### Cell Culture

Cell lines were from the American Type Culture Collection (Manassas, VA) or the Leibniz-Institute DSMZ Deutsche Sammlung von Mikroorganismen und Zellkulturen GmbH (Braunschweig, Germany), except for MUTU-I, an EBV^+^ Burkitt's lymphoma (BL) line, which was a gift from Dr. Alan Rickinson (University of Birmingham). These were validated through our Characterized Cell Line Core Facility, and grown in RPMI 1640 or Dulbecco's modified Eagle medium (Thermo Fisher Scientific; Waltham, MA) with 10% fetal bovine serum, penicillin, and streptomycin (Sigma-Aldrich).

### Immunoblotting, Enzyme-Linked Immunosorbent Assays, Immunoprecipitations

Protein expression was determined by immunoblot analysis as previously described (34). Antibodies used were sourced as follows: anti-HSP70-1 (C92F3A-5) and -HSF-1 were from Enzo Life Sciences, Inc. (Farmingdale, NY); anti-HSP70-1 (MA3009) was from Thermo Fisher Scientific; anti-td-Tomato was from MyBioSource, Inc. (San Diego, CA); and anti-p53, -HSP90, -HSP40, -CD9, -CD63, -LAMP-2, and -β-actin were from Santa Cruz Biotechnology, Inc. (Santa Cruz, CA). The anti-HSP70 IgG ELISA kit from Enzo Life Sciences, Inc., and the TeloTAGGG^TM^ Telomerase PCR ELISA from Roche (Basel, Switzerland) were used according to the manufacturer's instructions. RPMI 8226 cells were treated overnight with RTX or IVIgG (both at 5 mg/ml), and the culture supernatant was collected. Cells were then washed in PBS to remove these antibodies, lysed in IP Lysis buffer (Thermo Fisher Scientific), and both the harvested supernatant and the cell lysate was incubated with protein A/G agarose (Sigma-Aldrich). The agarose beads were incubated overnight at 4°C and washed in IP lysis buffer and immunoblotting was performed for HSP70-1.

### Plasmids and Quantitative Real-Time Polymerase Chain Reaction

Total RNA from treated cells was reverse transcribed as previously described (35). cDNA was subsequently used in quantitative real-time polymerase chain reaction (qPCR) for *HSP70-1* using inventoried TaqMan human *HSP70* probes and primers with *GAPDH* as a control (Thermo Fisher Scientific). The *HSP70* promoter construct was generated from MM1.S cell genomic DNA using the forward and reverse primers: TCGGGGTACCGCCTTTCAGGTTCACAATCAATCAG, which contains a KpnI restriction site and binds 695 base pairs (bps) upstream of the *HSP70* transcription initiation site; and GGCAAGATCTCAGGTTCGCTCTGGGAAGCCTTGG, which binds 87 bps downstream and has a BglII site. The resulting product was cloned into the pGL3 luciferase vector (Promega Corporation; Madison, WI). The palmitoylation (PALM) sequence tag coupled to the tdTomato fluorophore (tdT) vector was a gift from Xandra Breakefield (Massachusetts General Hospital).

### Cell Viability Assay and Flow Cytometry

The WST-1 reagent from Roche was used to determine cell viability, while apoptosis was determined by Annexin V Pacific Blue and TO-PRO-3 (Life Technologies; Grand Island, NY) staining, along with Count Bright Beads (Thermo Fisher Scientific), and analyzed by flow using FlowJo, version 10 (Tree Star, Inc.; Ashland, OR). Cell cycle analysis was performed using the NPE Analyzer^TM^ reagent (NPE Systems, Inc.; Pembrook Pines, FL) on a Gallios flow cytometer (Beckman Coulter, Inc.; Brea, CA), and analyzed using Multicycle Software (Phoenix Flow Systems; San Diego, CA). Live cells were treated with 1 μM ER-Tracker Green (Thermo Fisher Scientific) for 1 h at 37°C and washed in PBS prior to analysis with flow using the FITC channel.

### Gene Expression Profiling

The Illumina TotalPrep RNA Amplification Kit (Thermo Fisher Scientific) was used to generate amplified, biotinylated cRNA from total RNA isolated from OPM-2 cells treated with BSA or IVIgG in triplicate. The cRNA was hybridized overnight to Illumina HT-12 BeadArrays (San Diego, CA), stained with streptavidin-Cy3 (GE Healthcare; Amersham, PI), and scanned on a BeadArray Reader (Illumina). Bead-level data were extracted from GenomeStudio (Illumina) files and processed using open-source and custom software, following the approach of Dunning et al. (36). Values were corrected by the model-based background correction method that uses values for negative control probes to estimate and remove the non-specific signal component for each transcript probe, and the quantile distribution was normalized. We excluded the ~20% of probes that were not good matches to actual transcripts, and log_2_-transformed the probe-level data, which are the median values for each probe after discarding outliers. The relative gene expression in BSA and IVIgG samples were used to generate fold-change data, and *p*-values and heat maps were generated.

### Cytokine Analysis

The Bio-Plex Pro^TM^ human cytokine 48-plex screening panel (Bio-Rad; Hercules, CA) was used to analyze BSA- and IVIgG-treated culture media supernatants and analyzed on a Bio-Plex® 200/LX200.

### Extracellular Vesicle (EV) Isolation

Normal growth medium from 50 × 10^6^ cells was collected, washed, and re-plated in serum-free RPMI containing 1x insulin-transferrin-selenium-ethanolamine (Thermo Fisher Scientific). EVs were then isolated from the culture media using the ExoQuick-TC reagent (System Biosciences; Palo Alto, CA), and particle size and concentration were measured using the ZetaView® Nanoparticle Tracking Analyzer (Particle Metrix; Meerbusch, Germany). CD63 and CD9 were used for validation, as was the presence of HSP70-1 and LAMP-2. The RPMI 8226 drug-naïve cells (WT) and RPMI 8226 BZB-resistant (V10R) myeloma cells (37) were engineered to express a plasmid carrying the PALM tdT tag (38).

### Ethics Statement and Xenograft Studies

Experiments were performed under procedures and protocols approved by our Institutional Animal Care and Use Facility. Six- to 8-week-old C.B-17 severe combined immunodeficiency (SCID) mice (Envigo; Indianapolis, IN) (5 per group) were injected intravenously with 1 × 10^6^ luciferase-labeled MM1.S cells, and tumor burden was monitored by bioluminescent imaging using the IVIS Spectrum (PerkinElmer. Inc.; Waltham, MA). At 3 weeks post-cell injection, mice were randomized to once-weekly PBS or IVIgG (500 mg/kg) for 2 weeks, or intraperitoneal BZB (0.5 mg/kg) twice weekly for 2 weeks in 30% poly-(ethylene-glycol). Mice were sacrificed by CO_2_ asphyxiation when they displayed distress.

### Statistical Analyses

Data were analyzed by calculation of the standard error of the mean (SEM) and *t*-tests were used with a significance level of <0.05. The percentage change of tumor growth was plotted over time and the overall anti-tumor activity was the adjusted area under the curve (AUC) computed as an average AUC over time per mouse (39). Using both measurements, pairwise comparisons of the treatment groups were performed using the two-sample *t*-test and Wilcoxon rank sum test.

## Results

### IVIgG Induces Apoptosis and Cell Cycle Arrest

**Figure 1:**
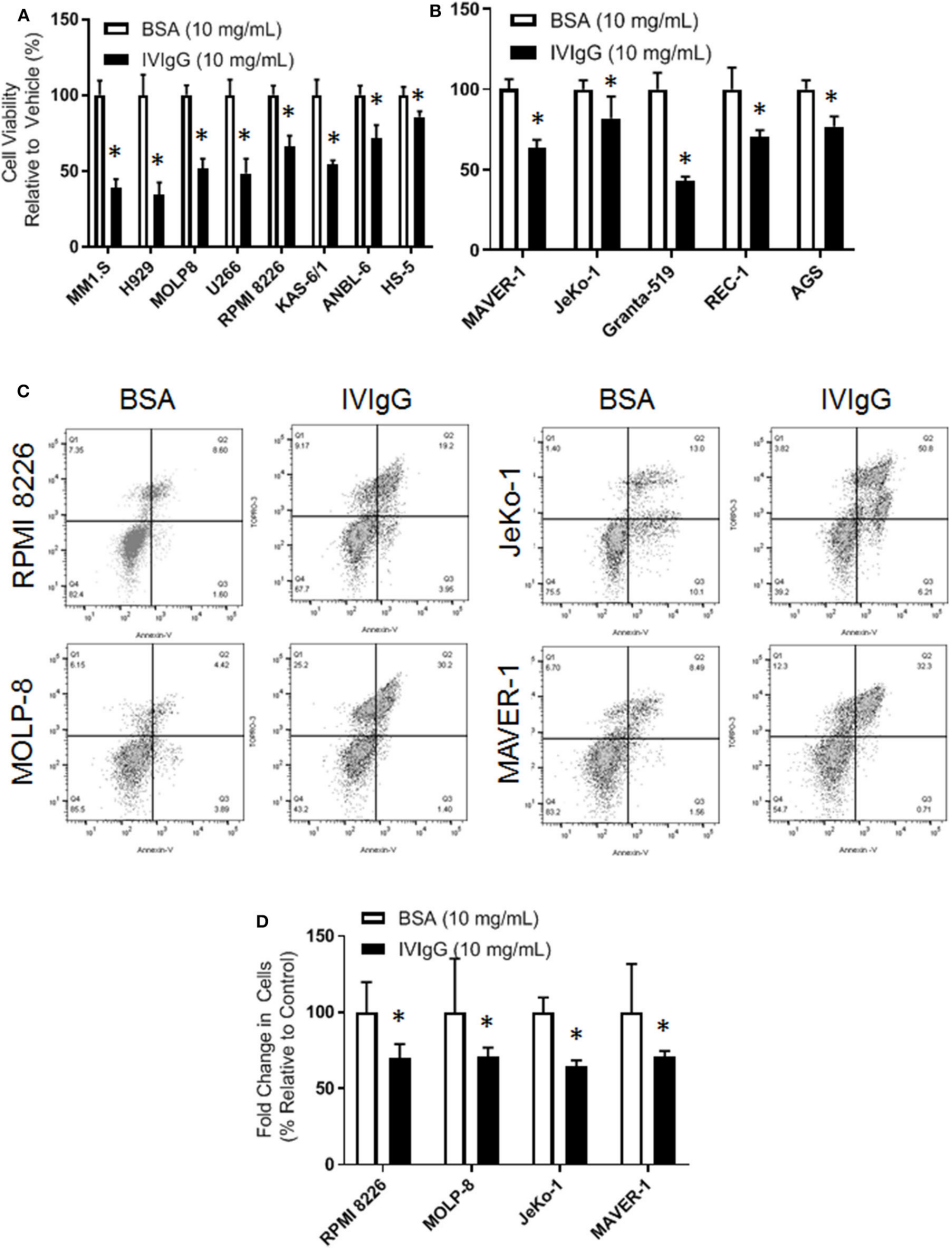
IVIgG inhibits growth of multiple myeloma and lymphoma cell lines. **(A)** Myeloma cell lines and the stromal cell line HS-5 were exposed for 72 h to IVIgG or bovine serum albumin (BSA), and cell viability was determined using the WST-1 assay. Results are expressed as the percentage viability relative to the vehicle control, which was arbitrarily set as 100%. **(B)** Cell viability of MCL cell lines and the Fc-gamma receptor-negative gastric cell line AGS after exposure to BSA vs. IVIgG was determined as above. **(C)** Flow cytometric analysis of RPMI 8226 and MOLP-8 cells, as well as of JeKo-1 and MAVER-1 cells exposed to BSA or IVIgG (10 mg/mL) for 72 h was performed after staining with Annexin V/TO-PRO-3. **(D)** Annexin V/TO-PRO-3 and Count Bright Bead flow was used to determine the change in viable cell number in response to BSA versus IVIgG. The values shown represent the mean ± standard error of the mean from three independent experiments, and an unpaired *t*-test was used to evaluate significance, where “*” denotes *p* < 0.05.

To begin to evaluate IVIgG's effects on myeloma and MCL cell lines, we exposed them to IVIgG or BSA, and used IVIgG at 10 mg/mL, which is equivalent to the IVIgG serum level achieved at the 2 g/kg dose for autoimmune disorders (40). Exposure of myeloma (Figure 1A) and MCL (Figure 1B) cell lines to IVIgG led to an anti-proliferative effect compared to the BSA control, with cell viability decreases ranging from 60% in MM1.S to 35% in ANBL-6 cells. Testing of marrow-derived HS-5 stromal cells and the gastric cancer cell line AGS, which do not express Fc receptors, demonstrated that IVIgG also reduced their viability (Figures 1A,B). Studies of RPMI 8226 and MOLP-8 myeloma, and of the JeKo-1 and MAVER-1 MCL cell lines, indicated IVIgG induced apoptosis based on the appearance of Annexin V-positivity (Figure 1C). Cell death occurred in 20% of RPMI 8226 and 30% of MOLP-8 cells, and 50% of JeKo-1 and 32% of MAVER-1 cells (Figure 1D). Cell cycle analysis in MOLP-8, U266, and HS-5 cells indicated IVIgG induced a G_1_S growth arrest (Supplementary Figure 1), though this was limited to a slight increase in the S phase fraction in MM1.S cells.

### Impact on Cytokines and Endoplasmic Reticulum (ER) Abundance

**Figure 2:**
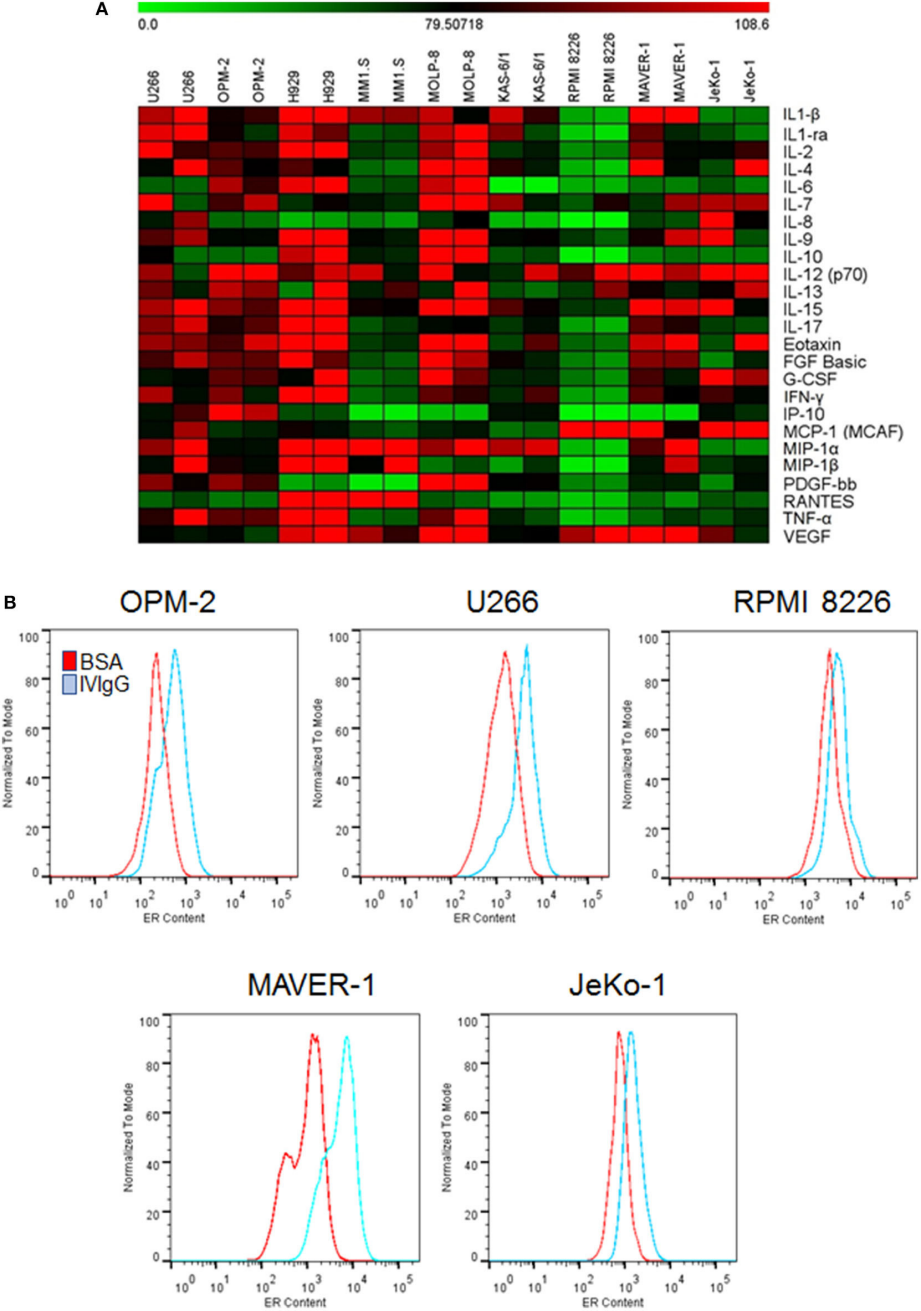
IVIgG-suppresses pro-myeloma and lymphoma cytokines and increases ER abundance. **(A)** The supernatant from IVIgG- and BSA-treated (10 mg/mL) MM or MCL cells was analyzed using the Bioplex human cytokine array in duplicate. Fold changes in expression were determined using BSA as a normalizing factor for the IVIgG-exposed samples. **(B)** MM and MCL cells were treated as above for 72 h, and live cells stained with ER-Tracker Green and analyzed by flow cytometry.

Given the known impact of IVIgG on immune-modulating cytokines, we evaluated its effect on MM and MCL cell line cytokine production. IVIgG induced pleiotropic changes in cytokines involved in immune regulation and growth (Figure 2; Supplementary Table 1). Most cells had decreases in Interleukin (IL)-6, Macrophage inflammatory protein (MIP)-1α, and five myeloma cell lines had a decrease in IL-8. In contrast, IVIgG stimulated the active heterodimer of IL-12 (IL-12-p70) in three myeloma and both MCL cell lines. Uptake of large amounts of IgG may lead to protein overload and expansion of ER abundance to cope with this excess protein (41). Myeloma and MCL cells treated with BSA vs. IVIgG, and stained with ER-Tracker Green, showed increased fluorescence, indicating an increase in the ER abundance (Figure 2B). Similar results were seen in BL cell lines (Supplementary Figure 2).

### IVIgG Suppresses HSP70-1 Expression

**Figure 3:**
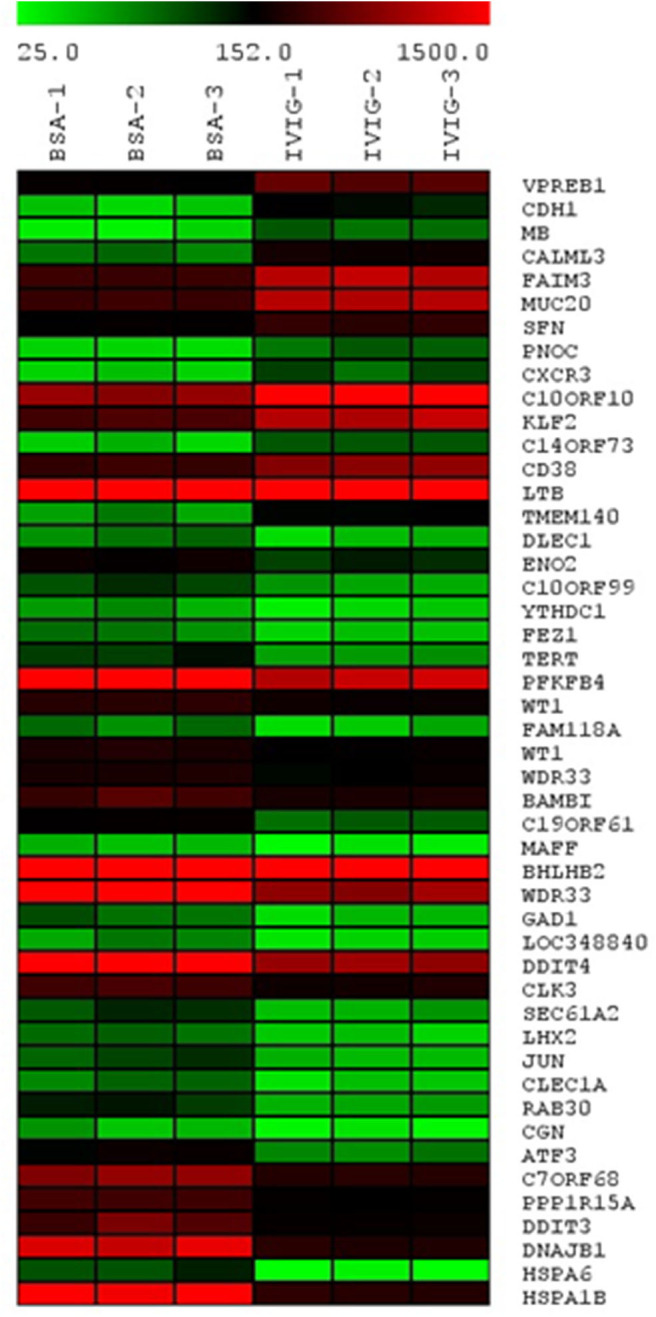
Transcription of HSP70 family members *HSPA1B* (HSP70-1), *HSPA6*, and *DNAJB1* is reduced by IVIgG. The OPM-2 myeloma cell line was exposed in triplicate to BSA or IVIgG (10 mg/mL) for 48 h, subjected to gene expression profiling (GEP), and the relative gene expression levels were organized in a heat map based on fold change compared to the BSA control. These cells were selected for GEP because they had a less robust cell death response to IVIgG at the time point used, allowing a focus on early transcriptional changes of IVIgG in the absence of later changes due to induction of apoptosis.

To gain a broader understanding of IVIgG's effects, we performed gene expression profiling of OPM-2 myeloma cells exposed to IVIgG or BSA. A 2-fold expression variance cut-off with a *p* < 0.03 identified 15 genes with increased expression, and 33 that decreased after IVIgG (Figure 3; Supplementary Table 2). The genes most stimulated included the early B-cell marker V-Set Pre-B cell surrogate light chain 1 (*VPREB1*; 3.67-fold change) and the tumor suppressor Cadherin 1 (*CDH1*; 2.88-fold). HSP70 family members were among the genes most suppressed, including *HSPA1B* (*HSP70-1*; 7.87-fold), its paralog *HSPA6* (*HSP70B*; 7.78-fold), and *DNAJB1* (*HSP40*; 6.85-fold). This coincided with decreased expression of DNA damage inducible transcript 3 (*DDIT3*; 6.85-fold), which is stimulated by HSP70-1 (42), and a decrease (4.14-fold) in Telomerase reverse transcriptase (*TERT*), another HSP70-1 associate (43, 44).

**Figure 4:**
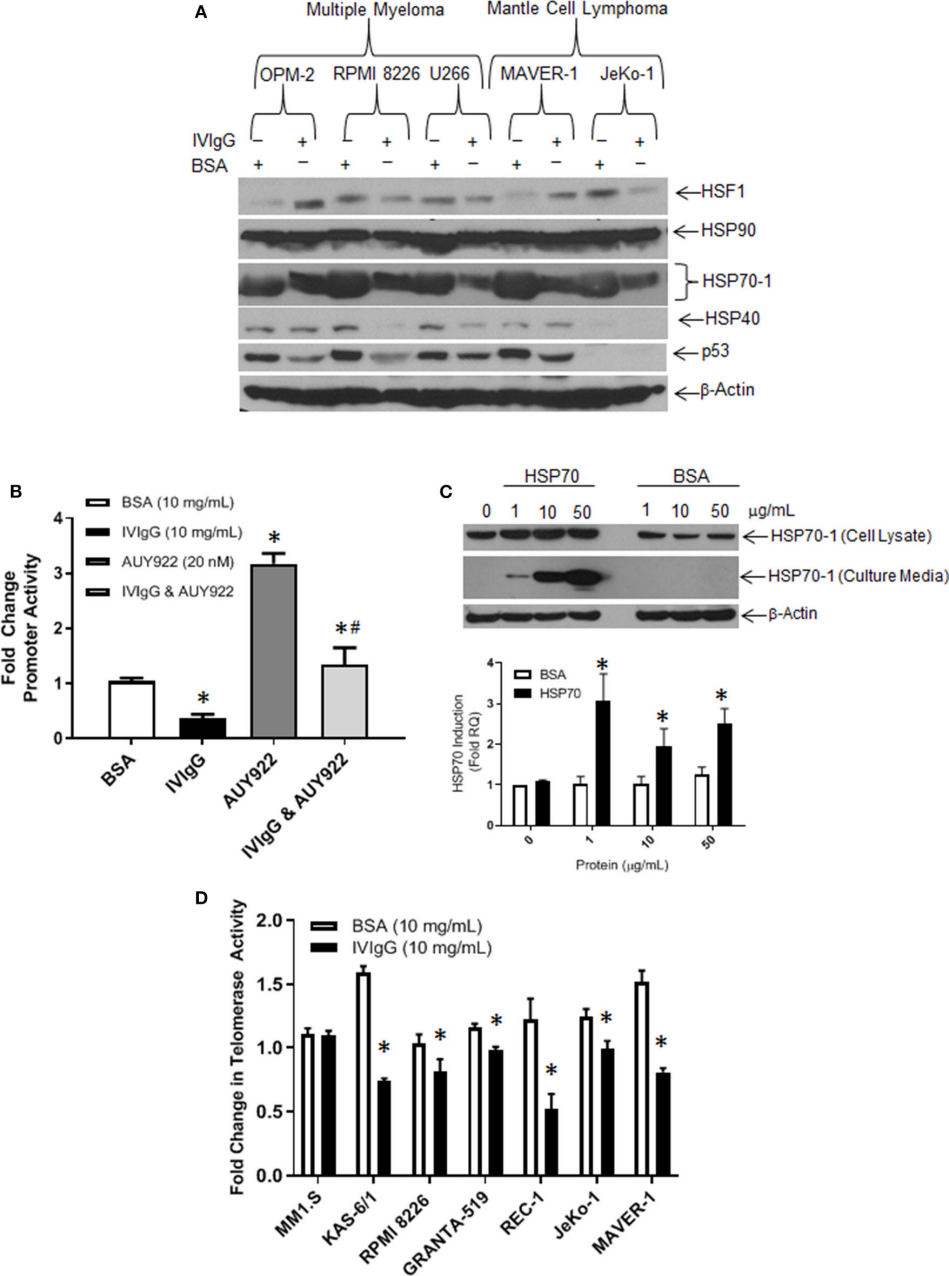
IVIgG suppresses expression of HSP70 and its co-chaperone protein targets. **(A**) MM and MCL cell lines were exposed to BSA or IVIgG for 48 h, and cell lysates were immunoblotted as indicated. **(B)** HEK 293T cells were transfected with an *HSP70-1* promoter luciferase reporter construct, and promoter activity was monitored following treatment with BSA, IVIgG, AUY922, or IVIgG and AUY922 for 24 h. Values were normalized to the BSA control (* denotes *p* < 0.05 compared to BSA and ^#^denotes *p* < 0.05 compared to AUY922). **(C)** RPMI 8226 cells were exposed to recombinant HSP70-1 for 24 h in serum-free media, and cell lysates and supernatants were evaluated with the indicated sera, while qPCR was performed on RNA extracted from cells and HSP70-1 transcription measured. **(D)** Telomerase activity in myeloma and MCL cell lines was evaluated using the TeloTAGGG^TM^ Telomerase PCR ELISA following 48 h of exposure to BSA or IVIgG, and fold change was calculated in relation to the BSA control. The values shown represent the mean ± standard error from three independent experiments, and an unpaired *t*-test was used to evaluate significance, where “*” denotes *p* < 0.05 compared to the BSA control.

Next, we evaluated the effect of IVIgG on HSP family members at the protein level. In all cell lines tested, HSP70-1 showed the largest change, and was lower with IVIgG in myeloma, MCL, and the BL models (Figure 4A; Supplementary Figure 3). Total levels of Heat shock transcription factor (HSF)-1, which induces *HSP70-1*, did not correlate with HSP70-1 since it was decreased in some cells but stable or increased in others, and HSP40 changes were similarly not consistent. The expression of p53, an HSP70 client (45), was also depleted in the majority, while HSP90 showed no change.

To further confirm the effect of IVIgG on *HSP70-1* transcription, we generated a luciferase reporter containing the *HSP70-1* promoter element. Transfected HEK 293T cells exposed to IVIgG vs. BSA showed a 50% decrease in basal promoter activity (Figure 4B). Addition of the HSP90 inhibitor AUY922 as a positive control increased *HSP70* promoter activity, while IVIgG suppressed this by 50%. Since HSP70-1 is secreted and can be taken up into a variety of cell types (46), we next used exogenous recombinant HSP70-1. Addition of 1 μg of HSP70-1 increased HSP70-1 levels in cell lysates (Figure 4C, top panel), and led to a significant (>3-fold) increase in *HSP70-1* transcription (Figure 4C, bottom). Given the known interaction between HSP70-1 and *TERT* (43, 44), we measured telomerase activity after IVIgG exposure, and five of six cell lines showed a decreased activity, with REC-1 and KAS-6/1 cells displaying the largest decreases of 50% (Figure 4D).

### Antibodies to HSP70-1 Are Present in IVIgG

**Figure 5:**
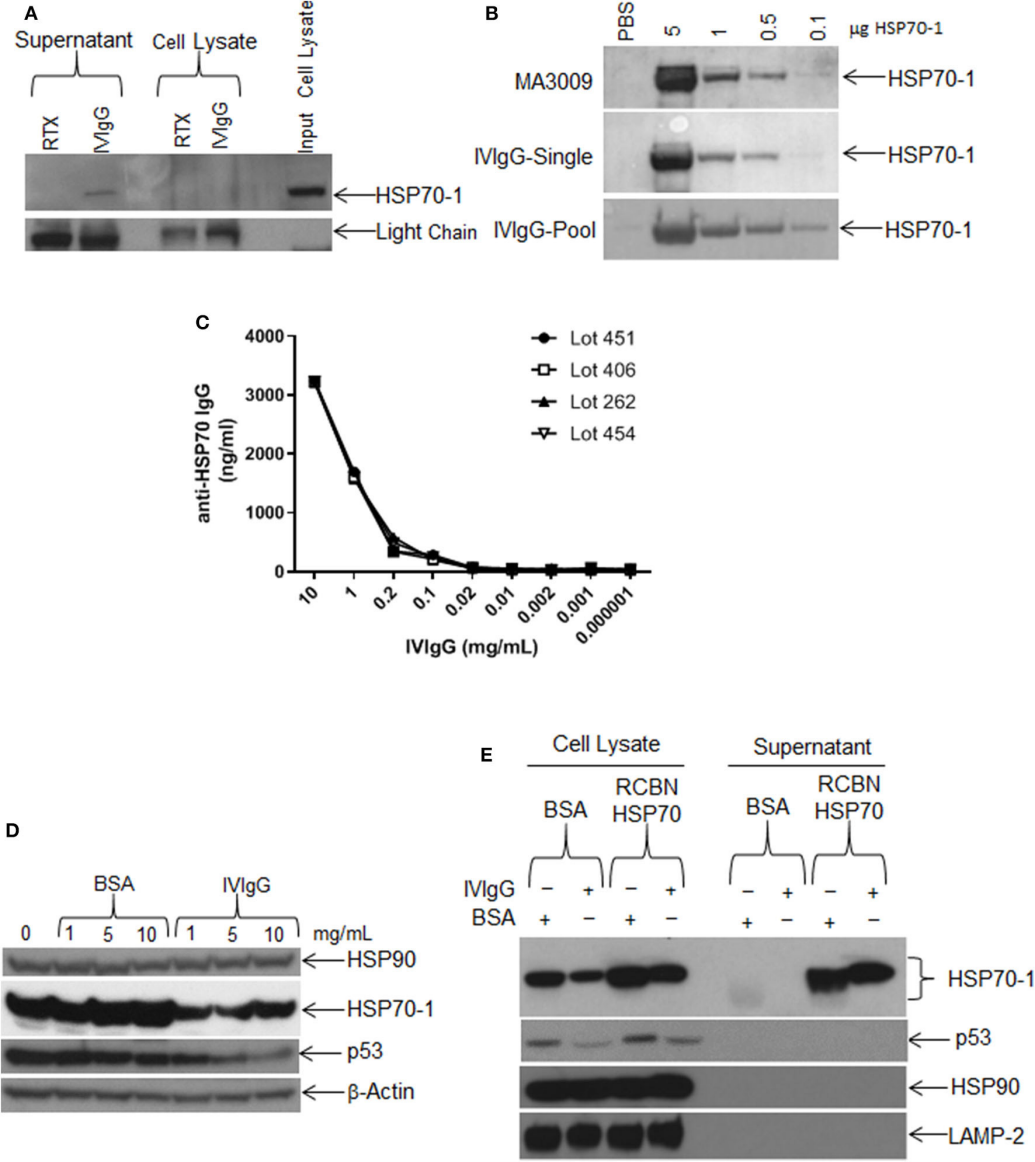
Single IVIgG lots contain high titers of anti-HSP70 IgG. **(A)** RPMI 8226 cells were treated for 24 h with rituximab (RTX) or IVIgG and culture media was immunoprecipitated with protein A/G agarose and immunoblotted for HSP70-1. **(B)** A single lot of IVIgG or multiple pooled lots were screened against increasing concentrations of recombinant HSP70-1 and compared to a commercially available anti-HSP70 IgG (MA3009). **(C)** Individual IVIgG lots were screened for anti-HSP70 IgG titers using a commercial ELISA and the concentration calculated for each lot. **(D)** RPMI 8226 cells were treated for 48 h with increasing concentrations of BSA vs. IVIgG and immunoblotted for the indicated targets. **(E)** RPMI 8226 cells were incubated for 24 h with BSA or IVIgG (10 mg/mL) in the presence of 50 μg of recombinant (RCBN) HSP70-1 and cell lysates of culture supernatant were immunoblotted as indicated.

In that extracellular HSP70-1 stimulated intracellular *HSP70A1A* transcription and enhanced protein abundance, we next evaluated if IVIgG could bind extracellular HSP70-1 shed from cells. Whole RPMI 8226 cells in culture were incubated with IVIgG or RTX as a control, and both the supernatant and intact cells were collected. Cells were then washed to remove IVIgG or RTX, and then the original culture supernatant and lysates of the intact cells unexposed to IVIgG or RTX were subjected to immunoprecipitation. IVIgG precipitated HSP70-1 from the cell culture supernatant, indicating both that IVIgG contains IgG against extracellular HSP70-1 and that the latter is shed into the media (Figure 5A). Recombinant HSP70-1 protein was then titrated onto a Western blot and immunoblotted using single IVIgG lots or a pooled mixture of lots, and IVIgG detected HSP70-1 (Figure 5B). Notably, screening of four different IVIgG lots using a quantitative anti-HSP70-IgG ELISA indicated the presence of anti-HSP70-1 IgG at ~3 μg/mL of IVIgG across the tested lots (Figure 5C; Supplementary Table 3).

We next titrated IVIgG onto cells to determine the dose effect on HSP70-1 levels compared to BSA. HSP70-1 decreased at the lowest IVIgG level used (1 mg/mL), and increasing this concentration did not suppress HSP70-1 further. In contrast, p53 levels were decreased at 5, and further decreased at 10 mg/mL, while there was no change in HSP90 or β-actin (Figure 5D). To see if the effect of IVIgG on HSP70-1 could be blocked, we added excess recombinant HSP70-1 to RPMI 8226 cells in the presence of IVIgG and evaluated cell lysates and supernatant. IVIgG reduced intracellular HSP70-1 to a lower level in the presence of BSA in the media than it could in the presence of recombinant HSP70 (Figure 5E). Moreover, the excess did not rescue the decrease in proliferation (data not shown), supporting the possibility that anti-HSP70 IgG within the IVIgG preparation bound exogenous HSP70-1 and modulated intracellular HSP70-1 levels.

### IVIgG Prevents Compensatory HSP70-1 Upregulation by HSP90 Inhibition

**Figure 6:**
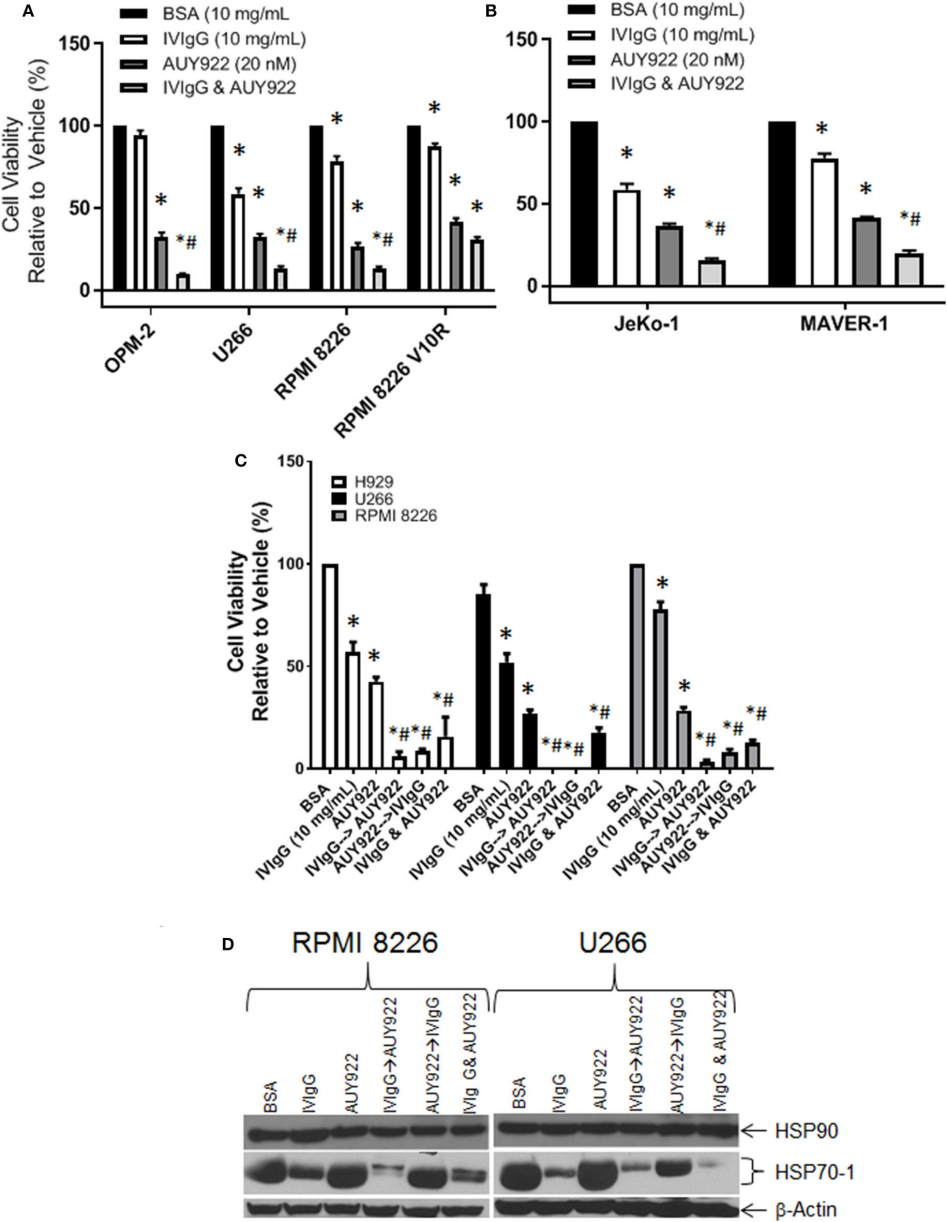
IVIgG suppresses AUY-922-mediated induction of HSP70-1 leading to enhanced cell death when used in sequence-specific combinations. Myeloma **(A)** and MCL **(B)** cell lines were treated with BSA, IVIgG, or AUY922 alone, or IVIgG with AUY922 for 72 h and cell viability was determined using the WST-1 assay. The BZB-resistant RPMI 8226 V10R cell line was also evaluated, along with its drug-naïve RPMI 8226 counterpart. **(C)** Cell viability measuring the sequence-specific effects of administration of IVIgG vs. AUY922 was then performed in myeloma cell lines, where IVIgG or AUY922 was added followed by the other agent 24 h later and then the two drugs were incubated for an additional 48 h. As a control, AUY922 and IVIgG were also added simultaneously. * denotes *p* < 0.05 to the BSA control and ^#^denotes *p* < 0.05 to both single agents. **(D)** Cell lysates from cells treated in **(C)** were evaluated for the indicated targets by immunoblotting.

HSP90 inhibitors are in trials for various malignances, and one mechanism of inducible chemoresistance is through upregulation of anti-apoptotic HSP70-1 (47). Treatment of OPM-2, U266, and RPMI 8226 cells with AUY922 decreased cell viability to ~30%, which was further decreased to 9, 15, and 13%, respectively, with IVIgG (Figure 6A), consistent with the possibility of an additive effect. The RPMI 8226 V10R BZB-resistant cell line exhibited resistance to IVIgG alone (87.25% viability) compared to drug-naïve RPMI 8226 cells (78% viability), and this was mirrored by a slight increase in resistance to AUY922, with viability dropping to 35% in V10R cells compared to 26% in the naïve cells. AUY922 and IVIgG showed limited benefit in V10R cells, with viability decreasing from 35% with the single agent to 30% with the combination (Figure 6A). Evaluation of two MCL cell lines revealed similar results: AUY922 decreased viability in JeKo-1 and MAVER-1 cells to 36 and 41%, respectively, which was further decreased to 15 and 19%, respectively, with IVIgG (Figure 6B).

Next, we evaluated whether adding the agents in a particular sequence optimized their effects. H929, U266, and RPMI 8226 cells all responded to IVIgG or AUY922, and simultaneous addition enhanced cell killing (Figure 6C). Using either IVIgG followed by AUY922, or the reverse, showed that IVIgG added first and AUY922 later generally produced a greater effect than AUY922 followed by IVIgG in H929 and RPMI 8226 cells. AUY922 stimulated HSP70-1 in U266 and RPMI 8226 cells (Figure 6D), while IVIgG followed by AUY922 precluded the AUY922-mediated HSP70-1 upregulation in RPMI 8226 cells, whereas AUY922 followed by IVIgG did not (Figure 6D). In U266 cells, IVIgG followed by AUY922, or with both agents simultaneously, led to suppression of AUY922-mediated HSP70-1 upregulation, whereas AUY922 followed by IVIgG did so to a lesser extent.

### IVIgG Enhances BZB Activity and Suppresses EV-Mediated Drug Resistance

**Figure 7:**
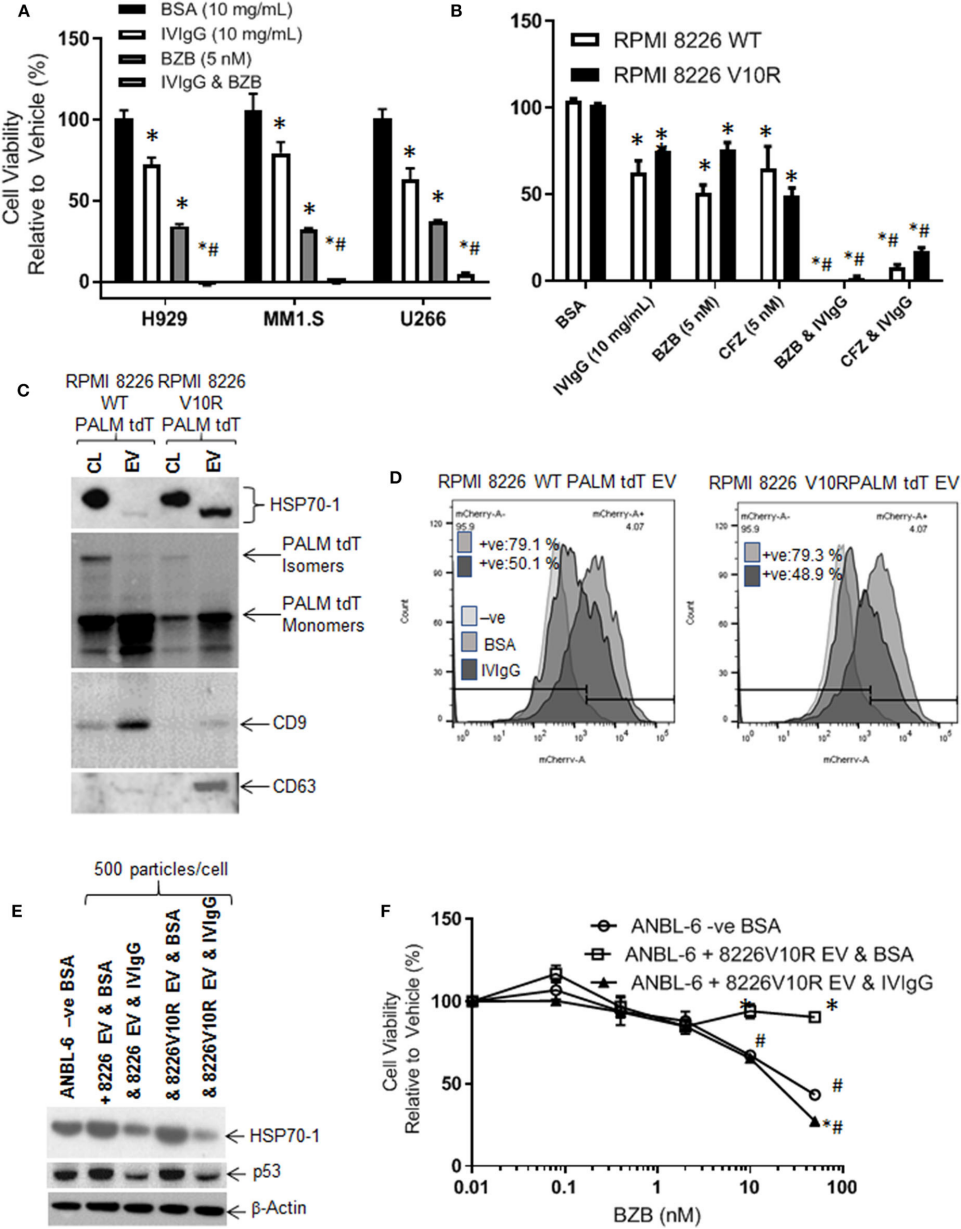
IVIgG prevents EV-mediated transfer of BZB resistance. **(A)** The anti-proliferative effect of IVIgG and BZB was evaluated in a panel of myeloma cell lines exposed to single agents or the combinations for 72 h followed by a WST-1 assay. **(B)** BZB-naïve RPMI 8826 and BZB-resistant RPMI 8226 V10R cells were exposed to single-agent BSA, IVIgG, CFZ, and BZB or a combination of IVIgG and either CFZ or BZB for 72 h, and the cell viability was measured by WST-1 assay. * denotes *p* < 0.05 compared to the BSA control and ^#^denotes *p* < 0.05 compared to both single agents. **(C)** EVs were isolated from BZB-naïve RPMI 8226 or BZB-resistant RPMI 8226 V10R cells carrying a PALM tdT tag to allow EV tracking, and EV lysates or the corresponding cell lysates (CL) were analyzed by immunoblot with the indicated antibodies. **(D)** ANBL-6 cells were exposed to EVs as described in **(C)** at 500 particles per cell for 48 h in the presence of BSA or IVIgG and analyzed for the percentage of PALM tdT tag-positive cells by flow cytometry. **(E)** Immunoblotting is shown of cell lysates from **(D)** probed as indicated. **(F)** Cell viability assay of ANBL-6 cells treated as in **(D)** with increasing concentrations of BZB for 48 h. ANBL-6 cells were utilized because they readily accepted EVs from RPMI 8226 cells in preliminary studies (not shown). * denotes *p* < 0.05 compared to ANBL-6 –ve BSA, whilst ^#^denotes *p* < 0.05 to ANBL-6 cells receiving 8226 V10R EV & IVIgG.

BZB is a key clinically relevant drug that is used for myeloma treatment in multiple settings but resistance frequently arises, and HSP70-1 expression has been associated with BZB resistance (48). We therefore evaluated the effects of IVIgG and BZB, and IVIgG alone reduced H929, MM1.S, and U266 cell viability by 28, 21, and 37%, respectively, BZB reduced viability by 66, 68, and 63%, respectively, and adding both led to virtually 100% cell killing (Figure 7A). Next, we examined the effect of IVIgG on RPMI 8226 wild-type (WT) and V10R cells alone and with BZB or CFZ. Wild-type RPMI 8226 cells were sensitive to IVIgG, BZB, and CFZ, with viability decreasing by 38, 50, and 35%, respectively, while IVIgG combinations with BZB or CFZ led to near complete cell viability loss (Figure 7B). Combining IVIgG with BZB or CFZ in the V10R cells led to enhanced loss of viability over the single agents alone, indicating that IVIgG enhanced BZB activity in cells previously resistant to the drug (Figure 7B).

HSP70-1 is found on the surface and inside EVs (49, 50), which may be drug resistance mediators. We therefore assessed IVIgG's effect on EVs from RPMI 8226 WT and V10R cells engineered to release PALM tdT-tagged EVs (38). EVs from WT and V10R cells displayed a 150 nM mean particle size for the WT and 110 nM for the V10R EVs (range for both, 50–800 nM; Supplementary Figures 4A,B). These EVs contained CD9 and CD63 tetraspanins (Figure 7C), while PALM tdT was detected in the cell lysates and EVs. Cell lysates had both smaller PALM tdT monomers and larger PALM tdT multimers, while EVs had enrichment of PALM tdT monomers only (Figure 7C). Interestingly, the V10R EVs showed enrichment of HSP70-1, whereas the WT EVs did not, and the HSP70-1 variant in cell lysates migrated as a slower form, while that found in EVs had an apparent lower mass.

As EVs could transfer HSP70-1 between cells, we were interested to see if IVIgG blocked this mechanism. We titrated 50–500 particles/cell onto ANBL-6 myeloma cells and found that 500 particles/cell gave 100% uptake (Supplementary Figure 4C). Next, we added WT- or V10R-derived EVs to ANBL-6 cells in the presence of BSA or IVIgG. EV uptake was highly efficient, with 79% of the ANBL-6 cells bearing the PALM tdT fluorophore from both the WT and V10R EVs. IVIgG addition suppressed this uptake to 50.1% in WT EV-treated cells and 48.9% in V10R EV-treated cells (Figure 7D). Lysates from the treated cells demonstrated that addition of WT and V10R EVs increased HSP70-1 and p53 levels compared to untreated cells, while IVIgG suppressed these to levels below the control cells (Figure 7E).

We also evaluated whether the V10R EVs could transfer BZB resistance to ANBL-6 cells. When these were incubated with 500 particles/cell and BZB was added with BSA or IVIgG, ANBL-6 control cells were sensitive to BZB but, with the addition of V10R EVs, they were resistant to the highest BZB concentration. Addition of IVIgG to the V10R EVs prevented the increase in resistance and restored BZB sensitivity to a level similar to the control cells (Figure 7F).

### Modeling *in vivo* Shows IVIgG Enhances BZB's Effects

**Figure 8:**
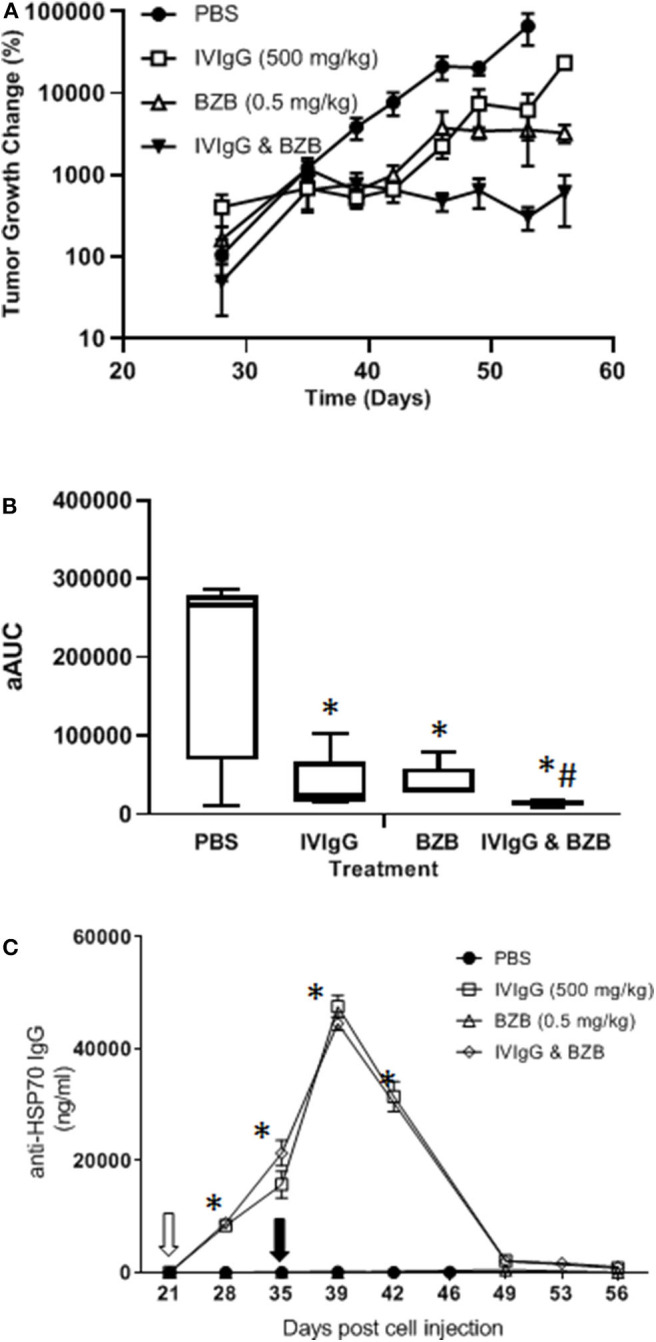
The IVIgG and BZB combination reduces myeloma disease burden and leads to high titers of anti-HSP70 IgG in mouse serum. **(A)** SCID mice injected with MM1.S cells carrying a luciferase expression cassette were monitored by IVIS imaging following a weekly dose of IVIgG for 3 weeks alone or IVIgG in combination with BZB twice per week for 2 weeks. The difference in tumor growth change (%) is shown. **(B)** Overall anti-tumor activity measured as the area under the curve (aAUC) over time is shown. **(C)** Serum titers of anti-HSP70 IgG in mice treated as above. * denotes *p* < 0.05 compared to the PBS control using the *T*-test in the above, while ^#^denotes *p* < 0.05 compared to the PBS control using the Wilcoxon rank sum test.

To evaluate whether IVIgG enhanced BZB's anti-tumor activity, we utilized a systemic model of myeloma in which human-derived, luciferase-labeled MM1.S myeloma cells hone to bone in immunodeficient SCID mice, which were treated with vehicle, IVIgG, BZB, or both. IVIgG or BZB suppressed MM1.S tumor growth (*p* < 0.05; *t*-test and Wilcoxon rank sum test) from days 39–49 (Figure 8A). Notably, IVIgG with BZB inhibited tumor growth beyond the single agents from day 40 to the conclusion of the experiment (day 56). In order to assess the overall anti-tumor activity, we calculated the average adjusted AUC (aAUC) over time per mouse. Both IVIgG and BZB as single agents reduced the aAUC, and the effect was greater with the two combined, with all treatments being significant (*p* < 0.05) (Figure 8B). Application of the Wilcoxon rank sum test demonstrated that only the combination had a significant effect when measured by the aAUC. Analysis of the serum from treated mice indicated that the IVIgG-containing regimens led to high anti-HSP70 IgG titers in the serum (~45 μg/mL at day 39), which was not seen with PBS or BZB (Figure 8C).

## Discussion

IVIgG has been used in cancer patients for immune maintenance following immune-suppressive therapies, but anecdotal reports detail remissions in a variety of malignancies. We evaluated the anti-cancer effects of IVIgG in myeloma and MCL cell lines, and found it decreased their proliferation and induced apoptosis, which coincided with cell cycle arrest (Figure 1; Supplementary Figure 1). Genomic analyses indicated a significant decrease in *HSP70-1* transcription and decreased HSP70-1 protein expression in multiple cell lines with decreased telomerase activity (Figures 3, 4D; Supplementary Table 2). While these two factors do not always correlate quantitatively, there is a qualitative concordance, and differences may be due to the varying half-lives of the corresponding mRNA and protein. Indeed, the half-life of *HSP70* mRNA is ~50 min (51), while that for HSP70 protein can be from 2 h (52) to up to 48 h or more (53) depending on the model and conditions studied. Interestingly, IVIgG suppressed activity of the endogenous *HSP70* promoter (Figure 4B), while exogenous HSP70 activated *HSP70* promoter activity (Figure 4C). It is tempting to speculate that these two are linked, and since extracellular HSP70 has been reported to induce Nuclear factor kappa B (NF-κB) in at least some models (54), and NF-κB can in turn up-regulate HSP70 (55), this may be the basis for such a positive feedback loop. However, additional studies will be needed to better understand this link, and it could be that IVIgG and HSP70 work on different pathways altogether.

Testing of multiple different IVIgG lots demonstrated a high titer of anti-HSP70 IgG, which recognized HSP70-1 by immunoblot and ELISA (Figure 5), and its effect on HSP70-1 was inhibited by exogenous HSP70-1 (Figure 5). A variety of chemotherapeutics induce HSP70-1, and this mechanism can help tumors circumvent pro-apoptotic effects (56). Notably, IVIgG suppressed HSP70-1 up-regulation by AUY922 when it was administered first, or with both together (Figure 6). Also, IVIgG with AUY922 or BZB enhanced anti-myeloma activity, and IVIgG enhanced BZB's activity against a BZB-resistant cell line (Figure 7). This sensitization occurred in association with an inhibition of transfer of EVs which themselves contained HSP70 (Figure 7). Finally, in an *in vivo* model, IVIgG with BZB were superior to the single agents, and increased anti-HSP70 IgG titers in mouse serum (Figure 8). It is possible that these *in vivo* effects of IVIgG could have been mediated in part through antibody-dependent cellular cytotoxicity by natural killer (NK) cells in the C.B17-SCID model. However, the mechanistic *in vitro* studies demonstrate that IVIgG can influence tumor cell sensitivity by influencing HSP70-1 levels in the absence of immune effector cells. It would also be interesting in future studies to examine cell surface HSP70-1 levels and how these are changed, if at all, by IVIgG, since tumor cells do express HSP70 (57, 58), and this can be a target for NK-mediated immune responses (59, 60).

One interesting aspect of our combination studies was that cells exposed to IVIgG first, and then an HSP90 inhibitor, suffered a greater loss in viability than if AUY922 was given first. IVIgG down-regulates HSP70-1 both by a direct mechanism, since it reduces cellular uptake of HSP70-1-containing EVs (Figure 7E), and an indirect mechanism by reducing *HSP70-1* transcription (Figure 4B). The latter is likely to be a slower mechanism since the half-life of *HSP70* mRNA is ~50 min (51). Therefore, if AUY922 is given first and strongly induces *HSP70* mRNA levels, then IVIgG is unable to as fully suppress HSP70-1 protein translation, leaving higher intracellular HSP70-1 levels (Figure 6D). This in turn allows cells to be more resistant to AUY922, producing a less robust reduction in cell viability, and arguing that, if this were ever to be a therapeutic strategy, pre-treatment with IVIgG should be prioritized.

IVIgG has previously been shown to have an array of effects in cancer, ranging from anti-inflammatory effects to the presence of specific antibodies that target proteins which impact tumor pathobiology. The spectrum of antibodies within IVIgG is reflective of normal serum from healthy individuals, and includes anti-idiotype antibodies to DNA, antibodies against TNF family members, Metalloproteinase-9 (61, 62), IL-6, IL-8, and Vascular endothelial growth factor (63). Indeed, in our experiments, IVIgG modulated several cytokines important for tumor cell growth (Figure 2; Supplementary Table 1). Several cancers, including pancreatic cancer, melanoma, sarcoma, and colon cancer, have been reported to respond to IVIgG in preclinical and clinical studies (27, 28, 64). Specific effects within each tumor type may depend on their antigenic burden or cytokine-dependence, and on the amount of anti-cancer IgG in the preparation. Immune modulatory effects of IVIgG may be mediated through Fc-gamma receptors, which are abundantly expressed on immune cells (65), and the *in vitro* effects of IVIgG often require a cross-linking antibody. However, we found that IVIgG's effect could be independent of the Fc-gamma receptor, as HS-5 stromal and AGS gastric cancer cell lines do not express these receptors (Figure 1).

Because HSP70-1 is abundantly expressed in many cancer types, our findings of high anti-HSP70 IgG titers within IVIgG is novel and potentially relevant. The presence of anti-HSP70-1 IgG in IVIgG indicates that healthy donors generate these antibodies to potentially modulate HSP70-1 function. Notably, HSP70-1 levels increase with pathogenic and normal health conditions, including after exercise and with cancer, preeclampsia, sickle cell disease, and septic shock (66, 67). Presence of anti-HSP70-1 IgG in these states represents a response to stress, while HSP70-1 protein levels in the circulation are decreased during normal pregnancy (68), which may decrease the mother's immune response to the fetus. Also of interest, anti-HSP70 IgG levels in expectant mothers were reportedly elevated in those whose developing children had fetal abnormalities and lower in those with normal fetuses (69). High HSP70-1 and anti-HSP70-1 IgG levels have also been reported in chronic spontaneous urticaria, which is associated with autoimmunity. Healthy donors had titers of anti-HSP70-1 IgG, IgM, and IgA at 53.36 μg/mL compared to 188.67 in the urticaria patients (70). The ability of the anti-HSP70 IgG in IVIgG to suppress HSP70-1 *in vitro* indicates that this could occur *in vivo* to control HSP70-1 levels, and the presence of anti-HSP70 IgG in IVIgG indicates that this could be a normal physiologic response. Higher levels of anti-HSP70 IgG in lung cancer patients have been shown to not correlate with increased HSP70 in the serum (67), and this could represent a breakdown in the immune response and a failure to elicit an increase in humoral immunity.

HSP70-1 is associated with drug resistance and is commonly found on EVs, including exosomes, microvesicles/ectosomes, oncosomes, and apoptotic bodies (50). This makes HSP70-1 especially interesting, particularly as its location on the EV surface can be used for purification (71) and therapeutically with antibodies or peptides (72). Our comparison of HSP70-1 expression in cell lysates and EVs indicated that the EVs contained a faster migrating HSP70-1 form (Figure 6C), suggesting that these EVs carry different cargo. Indeed, EVs may carry different/altered amounts of microRNA (73) compared to their producer cells and have different EV membrane protein cargo (74). The function of this faster migrating form remains to be determined, but its presence suggests it may have potential exocrine/paracrine effects. Our data showing that IVIgG reduced uptake of EVs into cells, and prevented transfer of BZB resistance, suggests this effect could be a result of the anti-HSP70 IgG in the preparation. We have not evaluated which mediators the EVs convey to recipient cells to transfer BZB resistance, but the proteasome subunits PSMA3 and PSMA3-S1 may transfer such resistance *in vitro*, and can be found in BZB-resistant myeloma patient EVs (75).

Our preclinical data may provide another potential facet to the growing list of therapeutic effects of IVIgG, and may help explain its activities against various tumors. The broad spectrum of IgG against various antigens in IVIgG precludes us from narrowing down the exact contribution of anti-HSP70 IgG, but we did demonstrate that exogenous HSP70-1 decreased suppression of HSP70-1 in treated cells (Figure 5E). Addition of IVIgG to the cancer armamentarium is an interesting idea, but may be precluded by its availability and cost, especially since these effects typically require the high, 1–2 g/kg dosing used in inflammatory disease. Use of HSP70-1 monoclonal antibodies (76) could be an alternative, since their greater specificity could allow for lower dosing and cost, though further studies would be needed to identify which epitopes are exposed and best targeted. Indeed, the recent interest in cancer immune therapy makes IVIgG a potential road map to identify new therapies that could be generated as monoclonal antibodies. It also suggests that a potential anti-HSP70 IgG-based therapy is unlikely to have significant side effects, given that high titers of such antibodies are already found in humans.

## Data Availability Statement

The datasets presented in this study can be found in online repositories. The names of the repository/repositories and accession number(s) can be found in the article/Supplementary Material.

## Ethics Statement

The animal study was reviewed and approved by MD Anderson Cancer Center IACUC.

## Author Contributions

RJ designed and performed most of the research and wrote the manuscript. RS designed and performed research and data analysis. FS performed the animal studies. JW and XW performed research. IK performed research, animal work, and cell studies. RD performed GEP. RO designed and supervised all the research completed herein and edited the manuscript. All authors contributed to the article and approved the submitted version.

## Conflict of Interest

RO declares research funding from BioTheryX, and has served on advisory boards for Amgen, Inc., Bristol-Myers Squibb, Celgene, EcoR1 Capital LLC, Forma Therapeutics, Genzyme, GSK Biologicals, Ionis Pharmaceuticals, Inc., Janssen Biotech, Juno Therapeutics, Kite Pharma, Legend Biotech USA, Molecular Partners, Sanofi-Aventis, Servier, and Takeda Pharmaceuticals North America, Inc., and is a Founder of Asylia Therapeutics, Inc., with an equity interest. RJ is a Founder of Asylia Therapeutics, Inc., with an equity interest. The remaining authors declare that the research was conducted in the absence of any commercial or financial relationships that could be construed as a potential conflict of interest.
