# Supplementary material for: Intravenous Immunoglobulin G Suppresses Heat Shock Protein (HSP)-70 Expression and Enhances the Activity of HSP90 and Proteasome Inhibitors
Source: Front Immunol. 2020 Aug 13;11:1816. doi: 10.3389/fimmu.2020.01816 (PMC7438474; doi:10.3389/fimmu.2020.01816)
Supplement: Supplementary file 1 [file Data_Sheet_1.PDF]

SUPPLEMENTARY FIGURE LEGENDS

Supplementary Figure 1. Treatment with IVIgG induces a G_1S cell cycle arrest.
The stromal cell line HS-5 and myeloma cell lines were treated with BSA or IVIgG for 72 hours, and the cell cycle distribution of the cells was analyzed using propidium iodide staining and flow cytometry. The percentages of cells in each stage of the cell cycle are shown.

Supplementary Figure 2. IVIgG induces accumulation of ER. BL cells were treated for 72 hours with BSA versus IVIgG, each at 10 mg/mL, stained with ER-Tracker Green, and analyzed by flow cytometry.

Supplementary Figure 3. IVIgG suppresses expression of HSP70 and its co-chaperone protein targets in BL cell lines.
BL cell lines were exposed to BSA or IVIgG for 48 hours, and cell lysates were immunoblotted with the indicated antibodies.

Supplementary Figure 4. EV size analysis of RPMI 8226 WT and BZB-resistant cells and titration into cells.
EVs from RPMI 8226 WT **(A)** and RPMI 8226 BZB-resistant **(B)** cells were analyzed using a ZetaView® NTA small particle analyzer to determine particle size and concentration. **(C)** ANBL-6 cells were incubated with increasing concentrations of EVs isolated from RPMI 8226 WT-PALM tdT cells and incubated for 24 hours and analyzed by flow cytometry to monitor percentage positive cell number.

SUPPLEMENTARY TABLES

Supplementary Table 1. Suppression of MM and MCL derived cytokines by IVIgG.
The supernatant from IVIgG- and BSA-treated (10 mg/mL) MM or MCL cells was analyzed using the Bioplex human cytokine array in duplicate. Fold changes in expression were determined using BSA as a normalizing factor for the IVIgG-exposed samples.

Supplementary Table 2. IVIgG significantly alters the expression of 48 different genes compared to the BSA control by GEP.
The OPM-2 myeloma cell line was exposed in triplicate to BSA or IVIgG (10 mg/mL) for 48 hours and subjected to GEP. The relative gene expression levels were determined by comparing BSA to IVIgG, the mean expression was calculated, and only genes with a two-fold or higher change with a p value <0.03 are shown.

Supplementary Table 3. Concentration of anti-HSP70-1 IgG in different lots of IVIgG.
Four randomly chosen IVIgG (Privagen) lots were evaluated for the titers of anti-HSP70-1 using a commercially available ELISA kit, and values are expressed in μg/mL.

Supplementary Figure 1

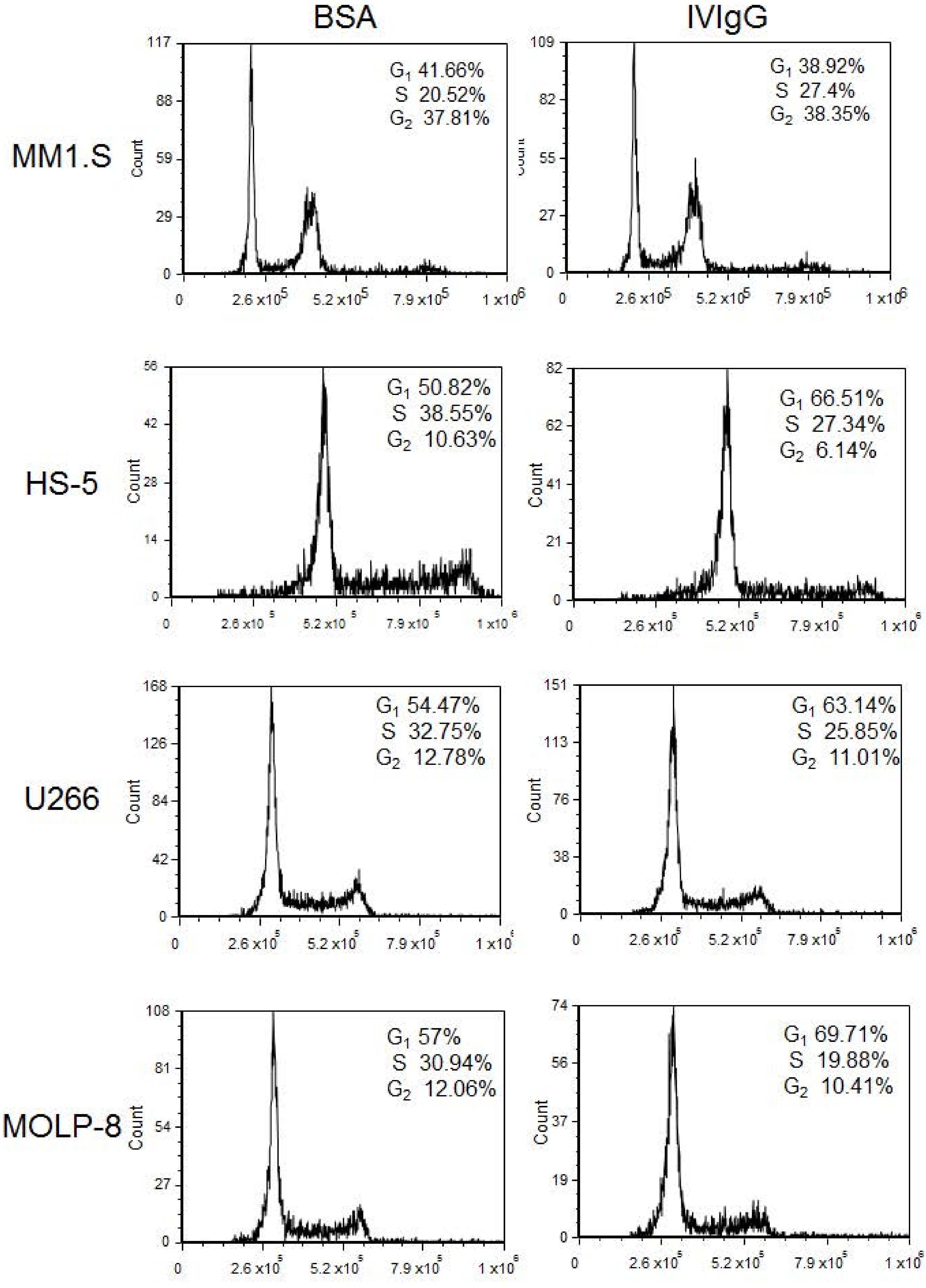

Supplementary Figure 2

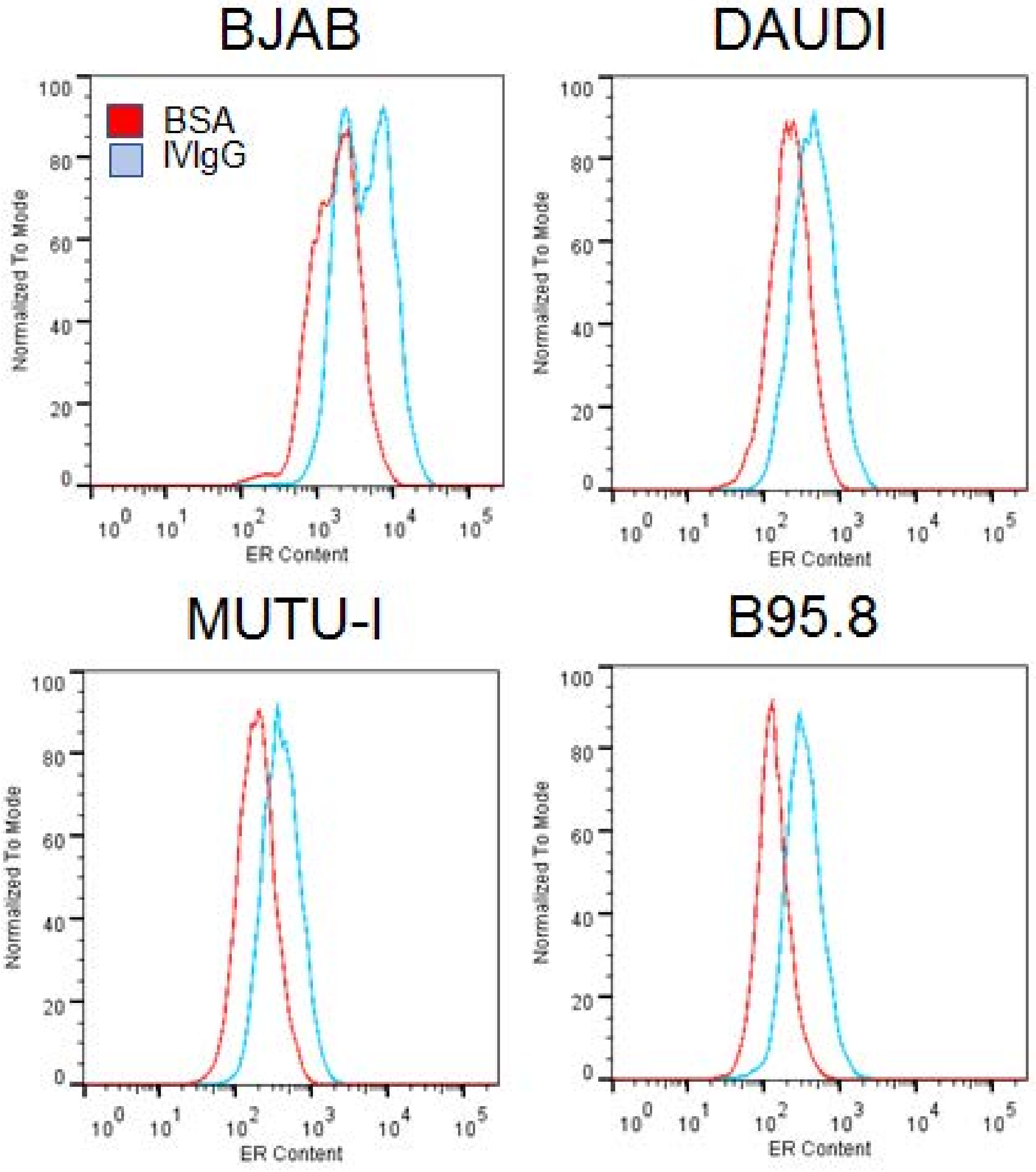

Supplementary Figure 3

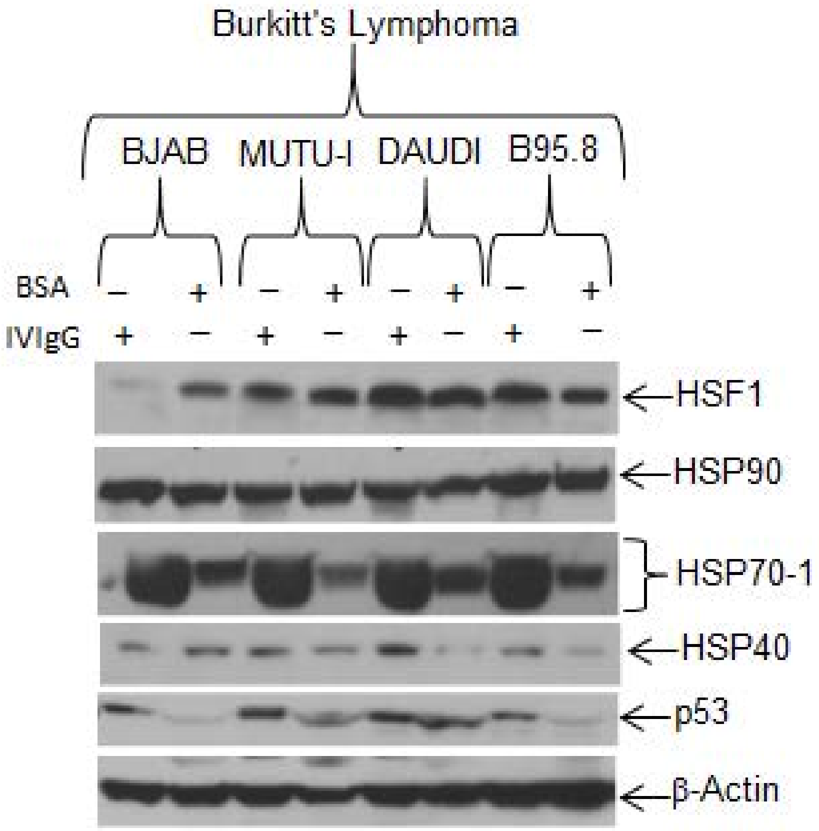

Supplementary Figure 4

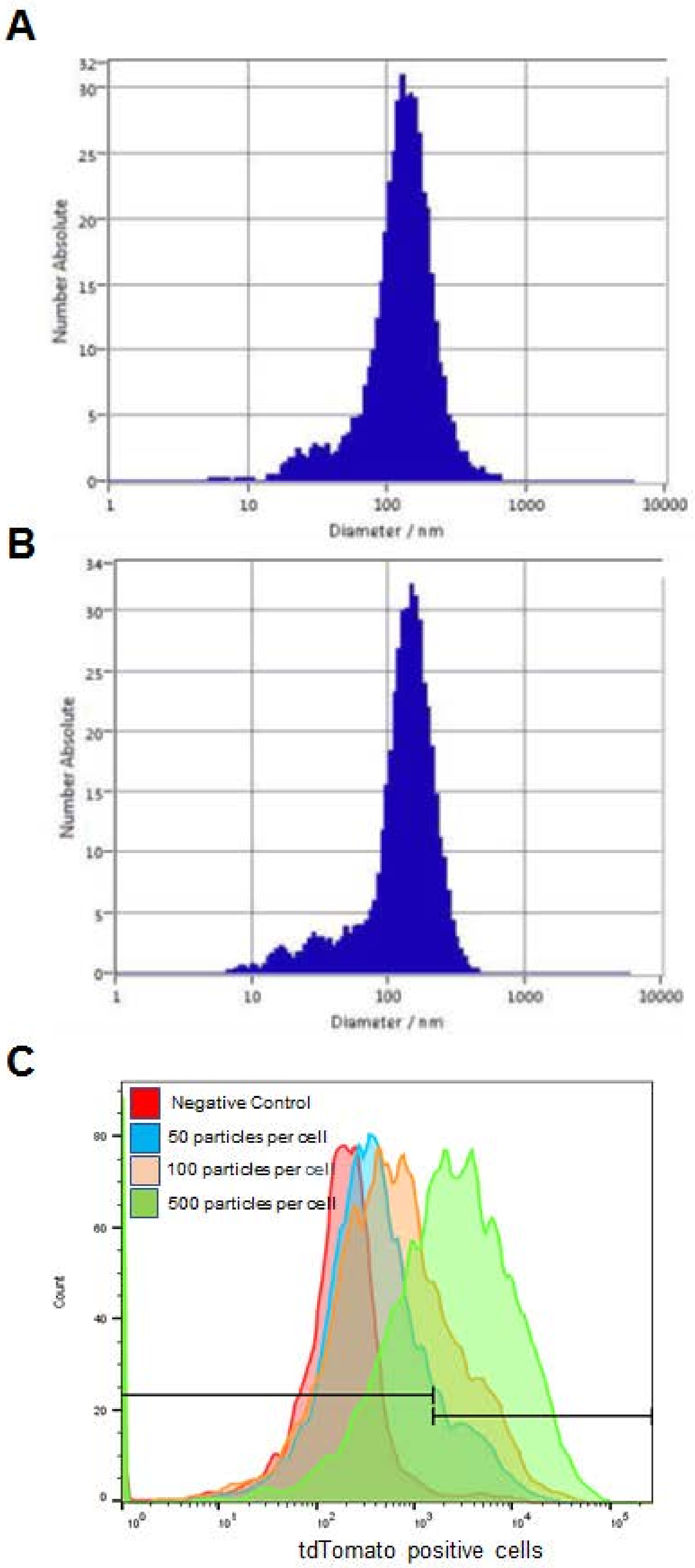

Supplementary Table 1.

	Fold change to control (%)								
Cytokine	U266	OPM-2	H929	MM1.s	MOLP-8	KAS-6	RPMI-8226	MAVER-1	JeKo-1
IL1-β	107.04	83.67	184.55	96.50	90.13	94.33	25.04	123.99	40.30
IL1-ra	111.52	72.39	98.52	54.96	105.34	75.96	13.04	79.37	48.70
IL-2	103.49	88.16	195.53	58.95	107.33	67.25	37.30	85.67	81.53
IL-4	98.96	84.77	83.72	44.57	126.31	78.08	20.38	115.28	84.52
IL-6	48.96	92.31	114.86	54.68	114.75	3.38	24.25	41.03	46.70
IL-7	100.18	94.08	72.66	69.45	126.59	84.51	67.03	80.32	99.46
IL-8	84.27	45.55	26.02	34.80	70.75	20.69	1.23	56.75	104.20
IL-9	93.72	77.07	146.44	74.19	117.56	78.64	55.88	92.79	81.10
IL-10	62.70	45.07	108.23	69.07	139.05	55.23	4.83	41.84	40.39
IL-12 (p70)	76.51	139.26	96.79	88.04	91.53	85.40	98.16	132.64	174.43
IL-13	80.78	98.52	96.85	78.81	108.62	49.98	77.73	74.72	91.72
IL-15	115.31	91.66	162.67	80.83	156.67	84.82	62.49	109.10	115.06
IL-17	99.76	86.17	118.96	57.81	77.77	68.82	26.41	86.72	58.12
Eotaxin	95.71	96.06	148.43	63.50	117.86	74.03	46.29	105.77	92.75
FGF Basic	94.26	90.91	113.21	52.58	109.93	75.44	36.40	94.43	53.51
G-CSF	75.17	89.38	102.49	49.43	126.59	68.50	38.34	79.37	103.76
IFN-γ	87.38	81.74	171.84	50.03	87.48	77.23	27.67	84.67	82.43
IP-10	80.51	133.78	55.78	6.61	20.27	74.85	2.35	10.39	70.33
MCP-1 (MCAF)	86.71	66.59	78.37	55.12	70.70	47.05	178.27	107.47	126.80
MIP-1α	112.89	75.25	404.55	118.77	108.23	102.43	19.30	103.35	36.62
MIP-1β	125.10	79.64	203.22	114.40	50.08	47.40	6.06	88.07	67.54
PDGF-bb	87.84	91.46	31.39	7.57	130.85	79.53	36.41	70.46	56.29
RANTES	53.60	42.91	135.92	250.97	49.87	31.58	17.44	39.38	52.29
TNF-α	96.82	89.86	165.02	49.43	101.73	62.48	19.06	54.42	60.64
VEGF	75.58	68.69	113.10	91.88	147.36	78.41	103.54	151.24	78.82

Supplementary Table 2.

Gene	Relative Expression						MEAN BSA	MEAN IVIg	p value BSA:IVIgG	Fold Change
	BSA-1	BSA-2	BSA-3	IVIgG-1	IVIgG-2	IVIgG-3				
VPREB1	189.60	173.51	153.62	688.58	589.90	615.90	172.24	631.46	0.0009	3.67
CDH1	56.00	43.73	54.22	163.00	146.64	133.04	51.31	147.56	0.0015	2.88
MB	33.63	30.43	45.97	109.37	95.07	98.52	36.68	100.99	0.0003	2.75
CALML3	93.76	101.26	84.09	260.44	205.60	234.24	93.04	233.43	0.0039	2.51
FAIM3	478.45	453.32	461.98	1117.77	1192.79	1092.63	464.58	1134.40	0.0006	2.44
MUC20	444.99	476.92	453.11	1114.42	1086.13	1106.88	458.34	1102.48	0.0000	2.41
SFN	153.52	184.32	175.50	418.39	366.60	408.20	171.11	397.73	0.0004	2.32
PNOC	46.67	45.94	42.27	92.31	108.46	105.05	44.96	101.94	0.0023	2.27
CXCR3	46.82	56.63	46.56	120.72	95.22	118.94	50.00	111.63	0.0046	2.23
C10ORF10	956.05	853.63	944.28	2026.23	1962.39	2085.32	917.99	2024.64	0.0000	2.21
KLF2	496.09	561.59	547.47	1108.31	1065.22	1205.10	535.05	1126.21	0.0006	2.10
C14ORF73	49.48	63.93	44.41	109.23	109.59	109.33	52.61	109.38	0.0052	2.08
CD38	410.96	471.59	436.35	839.98	890.92	917.73	439.63	882.87	0.0001	2.01
LTB	2840.76	2867.79	2415.48	5274.55	5513.83	5129.06	2708.01	5305.81	0.0001	1.96
TMEM140	71.60	92.13	68.26	150.31	149.92	152.18	77.33	150.80	0.0048	1.95
DLEC1	79.44	94.28	102.16	42.13	58.71	64.57	91.96	55.14	0.0088	-4.00
ENO2	224.92	182.04	240.63	119.42	138.94	129.57	215.86	129.31	0.0146	-4.01
C10ORF99	111.43	131.06	117.26	77.49	69.03	66.76	119.92	71.09	0.0022	-4.07
YTHDC1	74.72	84.66	62.61	32.71	45.23	53.49	73.99	43.81	0.0132	-4.08
FEZ1	98.74	94.74	79.36	46.70	57.61	55.85	90.94	53.39	0.0050	-4.13
TERT	122.49	120.70	145.06	71.58	75.18	80.95	129.42	75.90	0.0066	-4.14
PFKFB4	2088.14	2039.92	2005.74	1101.99	1210.92	1270.45	2044.60	1194.45	0.0004	-4.16
WT1	355.98	371.09	390.26	238.59	199.54	211.22	372.44	216.45	0.0003	-4.19

FAM118A	100.19	79.60	100.32	42.88	50.90	68.88	93.37	54.22	0.0098	-4.19
WT1	297.10	330.88	291.73	165.68	169.78	198.11	306.57	177.86	0.0007	-4.20
WDR33	283.62	288.16	313.07	148.34	153.98	207.52	294.95	169.94	0.0052	-4.24
BAMBI	433.87	616.79	495.72	277.66	291.05	305.42	515.46	291.38	0.0251	-4.35
C19ORF61	172.84	183.95	193.25	96.26	107.36	106.65	183.35	103.42	0.0004	-4.36
MAFF	64.32	56.35	60.14	26.90	40.06	34.65	60.27	33.87	0.0037	-4.38
BHLHB2	2799.84	2545.02	2882.45	1531.37	1516.36	1560.35	2742.44	1536.03	0.0032	-4.40
WDR33	1738.28	1577.47	1612.38	908.44	842.72	1008.94	1642.71	920.03	0.0002	-4.40
GAD1	114.17	95.62	94.42	42.92	61.15	61.40	101.40	55.16	0.0032	-4.56
LOC348840	68.19	93.67	86.51	37.94	46.98	49.78	82.79	44.90	0.0113	-4.58
DDIT4	1514.58	1976.50	1834.29	952.49	994.61	908.21	1775.12	951.77	0.0117	-4.64
CLK3	488.63	536.48	473.57	230.79	250.84	321.27	499.56	267.63	0.0017	-4.64
SEC61A2	107.22	134.20	128.62	57.69	63.03	77.49	123.34	66.07	0.0032	-4.64
LHX2	99.46	104.91	95.15	51.91	58.06	46.63	99.84	52.20	0.0002	-4.77
JUN	102.14	118.64	129.83	64.58	59.37	58.89	116.87	60.95	0.0081	-4.79
CLEC1A	84.54	103.26	103.43	39.49	57.57	53.79	97.08	50.29	0.0026	-4.82
RAB30	138.53	141.61	122.31	63.44	69.33	75.64	134.15	69.47	0.0010	-4.82
CGN	78.85	53.63	61.16	29.46	39.61	28.74	64.55	32.60	0.0168	-4.95
ATF3	149.69	219.66	195.81	85.23	81.50	95.53	188.39	87.42	0.0173	-5.36
C7ORF68	827.24	933.10	918.89	339.56	372.84	349.38	893.08	353.93	0.0010	-6.04
PPP1R15A	503.18	488.16	481.59	182.45	157.30	181.66	490.98	173.80	0.0000	-6.46
DDIT3	452.13	799.56	582.50	184.63	186.35	207.34	611.40	192.77	0.0266	-6.85
DNAJB1	1313.15	1193.04	1381.53	369.78	304.90	317.40	1295.90	330.69	0.0006	-7.45
HSPA6	112.09	111.12	137.13	24.22	31.59	24.22	120.11	26.68	0.0026	-7.78
HSPA1B	1830.18	1440.57	1887.64	397.59	354.38	345.62	1719.46	365.86	0.0049	-7.87

Supplementary Table 3.

IVIg Lot	anti-HSP70 IgG Titer (μg/mL)
451	3.24
406	3.22
262	3.20
454	3.23
